# Olfactory stimulation with multiple odorants prevents stress-induced cognitive and psychological alterations

**DOI:** 10.1093/braincomms/fcae390

**Published:** 2024-11-05

**Authors:** Bruno Bandiera, Francesca Natale, Marco Rinaudo, Raimondo Sollazzo, Matteo Spinelli, Salvatore Fusco, Claudio Grassi

**Affiliations:** Department of Neuroscience, Università Cattolica del Sacro Cuore, Rome 00168, Italy; Department of Neuroscience, Università Cattolica del Sacro Cuore, Rome 00168, Italy; Fondazione Policlinico Universitario ‘A. Gemelli’ IRCCS, Rome 00168, Italy; Department of Neuroscience, Università Cattolica del Sacro Cuore, Rome 00168, Italy; Fondazione Policlinico Universitario ‘A. Gemelli’ IRCCS, Rome 00168, Italy; Department of Neuroscience, Università Cattolica del Sacro Cuore, Rome 00168, Italy; Department of Neuroscience, Università Cattolica del Sacro Cuore, Rome 00168, Italy; Department of Biomedical Sciences, Università degli studi di Sassari, Sassari 07100, Italy; Department of Neuroscience, Università Cattolica del Sacro Cuore, Rome 00168, Italy; Fondazione Policlinico Universitario ‘A. Gemelli’ IRCCS, Rome 00168, Italy; Department of Neuroscience, Università Cattolica del Sacro Cuore, Rome 00168, Italy; Fondazione Policlinico Universitario ‘A. Gemelli’ IRCCS, Rome 00168, Italy

**Keywords:** stress, odorants, anxiety, memory, depression

## Abstract

Acute and chronic stress markedly affects behavior by triggering sympathetic nervous system activation and several hypothalamus-pituitary-adrenal-dependent responses. Brain regions of the limbic system are responsible for the regulation of stress response, and different reports have demonstrated that their activity can be influenced by olfactory stimuli. Here we report that, in mice exposed to acute restraint stress, olfactory stimulation employing a combination of three odorants, i.e. vanillin, limonene and green odor (trans-2-hexenal and cis-3-hexenol) decreased anxiety behavior, assessed in the elevated plus maze, and halted recognition and spatial memory deficits, as appraised in two different object recognition tasks. Of note, when applied singularly, the same odorants were unable to block the detrimental effects of stress. We also found that the multiple odorants stimulation prevented the development of depressive symptoms assessed by the sucrose splash test and forced swim test in an experimental model of depression, i.e. mice exposed to a chronic unpredictable stress paradigm, and reduced interleukin 1β levels in the prefrontal cortex of depressed mice. Collectively, our data indicate that olfactory stimulation counteracts the detrimental effects of acute and chronic stress on mood regulation and cognitive functions, thus representing a potential tool for the treatment of stress-induced disorders.

## Teaser

Bandiera and Natale *et al*. reported that olfactory stimulation using multiple odorants halts the detrimental effects of both acute and chronic stress on mood and cognition, revealing a tight connection between olfactory stimuli and inflammatory signaling in the brain.

## Graphical Abstract

**Graphical Abstract:**
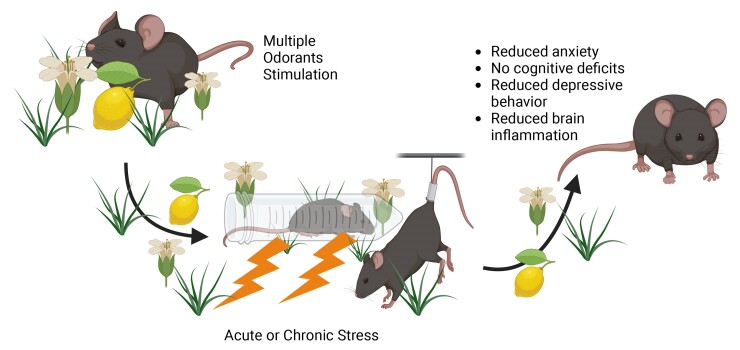

## Introduction

Environmental threats to physical and/or psychological integrity induce the activation of specific stress-related pathways allowing the individual to better respond or adapt to the source of stress.^1^ Stress-inducing stimuli activate the sympathetic nervous system and the hypothalamus-pituitary-adrenal axis, which in turn induce the production of stress hormones. The net effects of this complex nervous and hormonal response include increased heart rate and arterial blood pressure, glucose mobilization and increased respiratory rate, preparing the body for the fight or flight response.^2^ However, duration and intensity of the stressor can lead to different behavioral outcomes, rapidly precipitating their short-term beneficial effects to more pronounced long-term aberrations causing anxiety, cognitive alterations and increasing the risk for the development of mood disorders.^3^ The limbic system, an ensemble of cortical and subcortical brain regions including the medial prefrontal cortex, hippocampus, amygdala, and hypothalamus, is the main regulator of the stress response and it is highly influenced by sensory inputs, and especially olfactory stimuli.^4^ Olfactory information is first encoded within the olfactory bulb, which subsequently projects to the piriform cortex.^5^ From the piriform cortex, odorant-induced activity is then conveyed to amygdalar nuclei, the entorhinal cortex that is connected to hippocampus, to the prefrontal cortex and hypothalamus.^6,7^ A direct pathway connecting the olfactory bulb to the cortical amygdala has been reported to mediate innate odor-driven behavioral responses in mice.^8^ Direct projections from hippocampus to the anterior olfactory nucleus have been demonstrated to be necessary for the formation of odor-place associations.^9^ Olfactory inputs to the ventromedial hypothalamus also reduce the activity of corticotropin-releasing hormone neurons, which are activated in the presence of a stressor.^10^ Different reports have shown that olfactory stimuli can influence limbic activity, in terms of both stress response/emotional regulation and learning and memory processes in animal models as well as in humans.^11^

Aromatherapy has been employed by humans for centuries to improve different psychological functions.^12^ Indeed, some aromatic components derived from plants can modulate different receptors in the brain or they can, as well, stimulate olfactory perception. Different reports have shown that olfactory stimulation using particular odorants is able to impact on body physiology.^13^ In humans, it has been demonstrated that exposure to a pleasant odorant, such as black tea aroma, can reduce the levels of salivary chromogranin-A, which is used as a marker of stress levels,^14^ or lavender and rosemary smell induce an increase in free radical scavenging activity and reduce cortisol levels in saliva.^15^ Similar results have been obtained in animal models, where different odorants have been reported to modulate stress responses. In mice, exposure to roman chamomile essential oil combined with clomipramine improves behavioral symptoms in an experimental model of depression.^16^ Limonene inhalation reduces anxiety through the modulation of serotonin and dopaminergic receptors, whereas α-pinene can enhance the mRNA expression of neurotrophins.^17,18^ Other works have shown that green odor, a mixture of 3-trans-hexenal and 2-cis-hexenol, can improve anxiety symptoms in a mouse model of post-traumatic stress disorder while vanillin has been shown to possess antidepressant activity.^19,20^ However, most works have focused on stimulation with a single odorant molecule or essential oil, and a direct comparison between the effects of different odorants when inhaled singularly or presented in sequence has never been performed, nor an evaluation of their efficacy in halting stress-induced alterations on memory.

In this work, we sought to determine whether olfactory stimulation with a combination of different odorants could better impact and halt the detrimental effects of stress on mood and memory compared to the stimulation with a single odorant. We found that olfactory stimulation with a combination of vanillin, limonene and green odor prevented the development of anxiety, recognition and spatial memory deficits in a mouse model of acute restraint stress that we characterized at biochemical, molecular, and behavioral levels. The above-mentioned combinations of odorants also prevented the development of a depressive phenotype during exposure to chronic stress, by improving behavioral and molecular hallmarks of depression.

## Material and methods

### Animals

Wild-type C57BL/6 male mice (3–5 months of age), derived from the Animal Facility of Catholic University, were employed for this study. Mice were housed in groups of three to five animals per cage. The animals were kept at a controlled temperature of 24°C under a 12 h light/dark cycle with unrestricted access to food (Mucedola 4RF21, Milan, Italy) and water. Animals within the same litter were allocated to different groups.

### Ethics

All animal procedures were approved by the Ethics Committee of Università Cattolica and the Italian Ministry of Health (experimental protocol number 847/2021-PR). They were fully compliant with Italian (Legislative Decree No. 26/2014) and European Union (Directive No. 2010/63/UE) legislation on animal research. All efforts were made to limit the number of animals used and to minimize their suffering.

### Stress procedures and olfactory stimulation

Stress procedures were performed as previously reported.^21^ Briefly, for the acute restraint stress (ARS) procedure, animals were placed inside a 50 mL conic Falcon tubes for two hours and placed in their home cage. For the elevated plus maze, animals were allowed to recover for 30 min prior to the beginning of the test. For the novel object recognition and object place recognition, ARS was administered after the training phase. For experiments involving blood sampling and brain tissue harvesting, blood was collected immediately after the ARS procedure. At the end of blood collection animals were sacrificed to isolate the ventral hippocampus. For the chronic stress paradigm, the chronic unpredictable stress (CUS) was employed.^22^ Specifically, animals were subjected daily, for 6 weeks, to different stressors in an unpredictable fashion. Stressors employed were: 1) 3-h restraint stress; 2) 6 h food or water deprivation; 3) tilted cage at 45° for 6 h; 4) soiled bedding for 6 h; 5) tail pinch or tail suspension for 3 min; 6) cold water swim for 5 min; 7) cage with no beddings for 6 h. A couple of stressors were administered each day. Concerning the olfactory stimulation, the following odorants were employed: 1) vanillin, 1.8 mg/mL; 2) R-limonene, 10^−3^% v/v; 3) trans 2-hexen-1-al and cis 3 hexen-1-ol (green odor) at 10^−3^% v/v. All odorants were dissolved in 5% tween-aqueous solution and sprayed on the bedding of mice home cages. Mice subjected to only the ARS protocol received a total of 12 sprays (80–100 µL for each squirt) with vehicle solution, delivered at 20 min intervals during the 2 h of ARS procedure, for a total of 72 sprays. Animals exposed to olfactory stimulation received either 12 sprays of a single odorant (single-odorant condition) or 4 sprays of each odorant (Multiple odorants condition, M.O., for a total of 12 sprays administered in a random order) every 20 min during the 2 h ARS procedure. During the 2 h of ARS protocol, both groups received a total of 72 sprays in their home cage. Mice subjected to the CUS protocol were daily exposed to olfactory stimulation consisting of 4 sprays of each odorant (for a total of 12 sprays/day presented at 5 min intervals in a random order) directly on the bedding of their home cage for the whole duration of the CUS protocol. Nesting material was changed only during the weekly cage change. All the odorants were sprayed in the cage in the morning, between 09:00 and 11:00 a.m. for the CUS procedure, while they were sprayed for the whole duration of ARS procedure. Animals were housed in ventilated cage racks (IVS system, Tecniplast company) where the air enters and exits from each singular cage with its own unique set of pipes, so that each cage has its own ventilation system. In this way, the possibility of cross-exposure to other odorants beyond those employed was excluded. All stress procedures were performed under veterinary staff supervision to control for animal health.

### Behavioral paradigms

Behavioral tests were performed as previously reported,^22,23^ with slight modifications. All tests were performed by experimenters blind to treatment, from 9:00 a.m to 14:00 p.m and using the ANY-maze tracking system (Stoelting^TM^). Animals from different experimental conditions were tested sequentially. Briefly, for anxiety analysis, animals were placed on the elevated plus maze (EPM) and allowed to explore the apparatus for 10 min. Time spent in the open and closed arms was recorded, as well as open arms entries. For the novel object recognition (NOR) and object place recognition (OPR) paradigms, animals were allowed to explore two identical objects placed within a square arena (33 × 33 cm) for 10 min. Twenty-four hours later, in the case of the NOR test, one of the objects was substituted with a novel one and animals were allowed to explore the arena and the objects for 5 min. For the OPR, instead, one of the objects was moved to a different position in the arena. Time spent exploring the novel/displaced object to total exploration time was then reported as preference index (PI) and as discrimination index (D.I., new/displaced minus old/stationary divided by total exploration time). For the EPM, NOR and OPR procedures, different cohorts of mice were employed. Between each animal, all the apparatuses and objects were cleaned with 70% ethanol. For the chronic stress procedure, the same cohort of mice was evaluated using the forced swim test (FST) and the sucrose splash test (SST), to measure stress-coping response and apathic/anhedonic behavior. For the FST, animals were placed in a baker filled with water at a temperature of 25° ± 1°C for six minutes, and total immobility time was recorded. Baker size was of 16 cm height × 12 cm diameter. Water depth was of 10 cm, leaving 6 cm from water surface to baker borders. All animals were tested using the same apparatus. The water was changed every 10 animals to avoid temperature shifts. Concerning the SST, animals were sprayed on their coat with 7 squirts of a 10% sucrose solution and grooming time was recorded for the following 5 min. For the open field test assessing locomotor activity in the chronic stress model, animals were placed inside a 45 × 45 cm square arena and allowed to explore for 10 min.

### ELISA

For corticosterone measurements the ELISA kit ADI-900-097 from Enzo life sciences was employed. Briefly, blood was collected, in the presence of EDTA, from the submandibular vein at the end of the ARS procedure. Samples were then centrifuged at room temperature at 600 g for 10 min, and plasma was subsequently isolated and stored at −80°C for the analysis. ELISA assay was then performed according to the manufacturer instructions. For the BDNF assay (kit IK-10146, Immunological Sciences) and IL 1β ELISA (kit number IK-4205, Immunological Sciences) the measurements were performed following manufacturer instructions and as previously reported.^24,25^ A total of 15 mice (n = 6 control mice, n = 6 CUS mice, n = 3 CUS + M.O.) were used for both BDNF and IL 1β dosage by ELISA.

### Western blot

Western blot analyses were performed as previously described.^23^ After ARS exposure, animals were sacrificed through cervical dislocation for brain tissues collection. Briefly, the brain was placed inside a brain matrix and sliced coronally at 4 mm from the olfactory bulbs, to collect the prefrontal cortex from anterior slices. Then, the ventral hippocampus was isolated as reported in,^26^ with slight modifications. The tissues were lysed in ice-cold lysis buffer (150 mM NaCl, 50 mM pH 8 Tris-HCl, and 2 mM EDTA) containing 1% Triton X-100, 0.1% sodium dodecyl sulfate, 1 × protease inhibitor cocktail (P8340, Sigma-Aldrich, Saint Louis, MO, USA), 1 mM sodium orthovanadate (S6508, Sigma-Aldrich, Saint Louis, MO, USA), and 1 mM sodium fluoride (201154, Sigma-Aldrich, Saint Louis, MO, USA). After lysis, tissues were spun down at 22,000× g and 4°C, and the supernatants were quantified for protein content (500006, DC protein assay; Bio-Rad, Hercules, CA, USA). Equal amounts of protein were diluted in Laemmli buffer, boiled, and resolved by SDS-PAGE. The primary antibodies were incubated overnight at 4°C and revealed with HRP-conjugated secondary antibodies (#7074 and #7076, Cell Signaling Technology Inc., Danvers, MA, USA). Primary antibodies for phospho-ERK1/2 and total ERK1/2 (catalogue references, respectively: #9101 and #9102 from Cell Signaling Technology Inc.), pNF-kB (AB11226, Immunological sciences), total NF-kB (ab16502, Abcam), PSD-95 (#3450, Cell Signaling Technology Inc.), pGSK3β (#9336, Cell Signaling Technology Inc.) and total GSK3β (#12456, Cell Signaling Technology Inc.) were diluted 1:1000. Primary antibodies for actin (ab8227, Abcam) and Hsp90 (#4877, Cell Signaling) were diluted at 1:10,000. Changes in protein phosphorylation were evaluated and documented using UVItec Cambridge Alliance (Uvitec, Cambridge, UK). Images shown were cropped for presentation without any other manipulation (see Supplementary materials for uncropped images). A total of 22 mice (n = 9 control mice, n = 8 CUS mice, n = 5 ARS mice) were used for western blot analyses.

### Immunofluorescence experiments

For immunofluorescent labeling, sections were processed as previously described.^27^ Animals were intraperitoneally injected with bromodeoxyuridine (BrdU; Sigma, St. Louis, MO, USA; 100 mg/kg dissolved in 0.9% NaCl solution) for the last 5 days of CUS exposure. Animals were deeply anesthetized and were transcardially perfused with PBS (0.1 M, pH 7.4) followed by 4% PFA. Brains were collected, post-fixed overnight at 4°C in PFA, and then transferred to a solution of 30% sucrose in 0.1 M PBS. Coronal brain sections (30-μm-thick) were cut with a vibratome (VT1000S, Leica Microsystems, GmbH, Wetzlar, Germany). Hippocampal slices were incubated sequentially with 2N HCl for DNA hydrolysis and epitope retrieval, 1×PBS with 0.3% Triton X-100 (Sigma, St. Louis, MO, USA) and 5% NGS at RT for permeabilization and blocking and then with BrdU antibody (1:500; Abcam, ab6362), overnight at 4°C. The next day, tissues were incubated for 90 min at RT with the secondary antibody: Alexa Fluor-488 anti-rat (1:600; Invitrogen, a11006). Finally, nuclei were counterstained with DAPI (0.5 μg/mL for 10 min; Invitrogen), and slices were coverslipped with ProLong Gold anti-fade reagent. Images (1024 × 1024 pixels) were acquired at 20× magnification with a Nikon A1 MP confocal system (Tokyo, Japan). For analyses, DAPI+/BrdU+, cells were counted. During brain sectioning, hippocampal slices were placed in a 6-wells plate, by sequentially adding 1 slice per well from the anterior part of the hippocampus (−1.70 mm from bregma) to the posterior (−3.16 mm from bregma). Every 6 slices, this procedure was repeated starting from the first well. Then, a single well, containing 8 slices, was employed for the analysis of the number of proliferating cells. All labeled cells within the subgranular zone (SGZ) of the dentate gyrus of each slice were counted separately. The sum of proliferating cells counted in 8 slices was then multiplied by 6 to estimate the total number of labeled cells in the whole hippocampus. Image acquisition and analysis were carried out using the software NIS Elements AR 5.30.01. A z-stack analysis, which allows to evaluate the fluorescence intensity of BrdU signal cell-by-cell along the 3 cross-sections XY, XZ, and YZ, was then performed. A total of 7 mice (n = 3 control mice, n = 4 CUS mice) were used for histological analyses.

## Statistical analyses

Sample sizes were calculated with adequate power (0.8) based on pilot studies and literature data. All statistical analyses were performed by using SigmaPlot 14 software (Systat Software, Palo Alto, CA, USA). Data distribution was first evaluated for equal variance and normality (Shapiro–Wilk test). All statistical tests used (Student’s *t* test, one-way ANOVA, one-way ANOVA on Ranks, two-way ANOVA and post-hoc tests) are reported in the main text. The sample sizes (n) are reported in the figure legends. Significance was set at 0.05, and all tests were two-tailed. The results are reported as means ± sem. No animals were excluded from the study.

## Results

### ARS induces a stress phenotype

**Figure 1:**
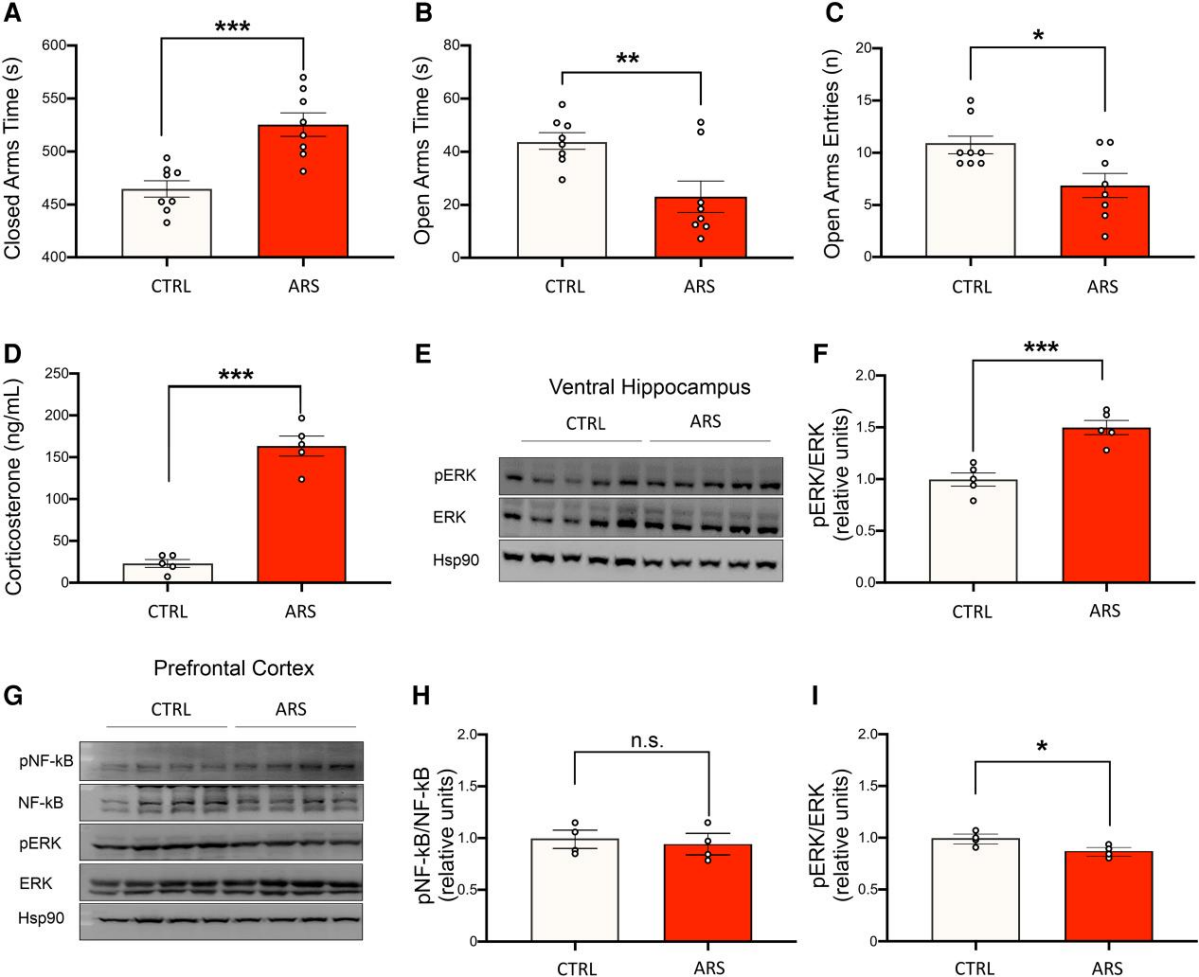
**Acute restraint stress exposed animals show behavioral, molecular, and biochemical hallmarks of acute stress response.** Animals were subjected to 2 h acute restraint stress (ARS). **A**, **B** and **C**) Thirty minutes after the end of the ARS protocol animals underwent the EPM paradigm. ARS-exposed animals showed a significant increase in time spent in closed arms (**A**) and a significant decrease in time and entries into the open arms (**B** and **C**, n = 8 for both groups). **D**) Corticosterone levels after ARS exposure. ARS-exposed animals show a significant increase in blood corticosterone levels compared to control animals (n = 5 for both groups). **E** and **F**) ERK phosphorylation at residues Thr202/Tyr204 was significantly higher in ARS-exposed animals (n = 5 for both groups). **G**, **H** and **I**) NF-kB activatory phosphorylation at Ser311 and ERK phosphorylation at residues Thr202/Tyr204 in the PFC of CTRL and ARS-exposed mice. No differences were observed in NF-kB phosphorylation while a statistically significant decrease in ERK phosphorylation was observed (n = 4 for both groups). All data are reported as mean ± s.e.m. Dots represent the number of samples (studied animals). Student’s *t* test for all comparisons; *P < 0.05; **P < 0.01; ***P < 0.001. See supplementary materials for uncropped blots.

To characterize the stress response in our experimental model of acute stress, we performed biochemical, molecular and behavioral analyses in mice subjected to ARS for 2 h. Thirty minutes after the end of the ARS procedure, animals were tested in the Elevated Plus Maze (EPM). ARS-exposed animals showed a significant increase in time spent in closed arms compared to control mice (525.0 ± 11.8 s versus 464.0 ± 8.2 s, respectively; Student’s *t*-test, t = 4.502 *P* < 0.001, Fig. 1A) and a significant decrease in both time spent in open arms (23.0 ± 6.3 s versus 44.0 ± 3.4 s; Student’s *t*-test, t = 3.132, *P* = 0.016; Fig. 1B) and number of open arms entries (6.8 ± 1.2 versus 10.7 ± 0.9; Student’s *t*-test, t = 2.711, *P* = 0.007, Fig. 1C). To avoid any possible influence of either EPM testing on the studied molecular parameters or of the blood collection on behavioral assessment, a different cohort of mice exposed to the same ARS protocol was employed for biochemical experiments. Blood was collected soon after the end of the 2-hour ARS procedure. Corticosterone levels in ARS-exposed animals were significantly higher compared to control animals (22.9 ± 5.28 versus 163.4 ± 13.4 ng/mL, respectively; Student’s *t*-test, t = 10.880, *P* < 0.001; Fig. 1D). After blood collection, animals were sacrificed, and the ventral hippocampus and prefrontal cortex (PFC) were isolated for western blot analyses. A significant increase of the extracellular signal-regulated kinases (ERK) activation was observed in the ventral hippocampus of ARS-exposed animals (43 ± 0.1% increase in ARS-exposed animals compared to control animals; Student’s *t*-test, t = 5.345, *P* < 0.001; Fig. 1E and F). In the PFC no significant difference in nuclear factor kappa B (NF-kB) activatory phosphorylation was observed between the two groups (5% decrease in ARS-exposed animals compared to control animals; Student’s *t* test, t = 0.416, *P* = 0.691; Fig. 1G and H), whereas a significant decrease in ERK phosphorylation was found in ARS-exposed mice (13% decrease in ARS-exposed animals compared to control animals; Student’s *t* test, t = 2.457, *P* = 0.049; Fig. 1G and I).

### Olfactory stimulation with multiple odorants halts the effects of ARS on anxious behavior

**Figure 2:**
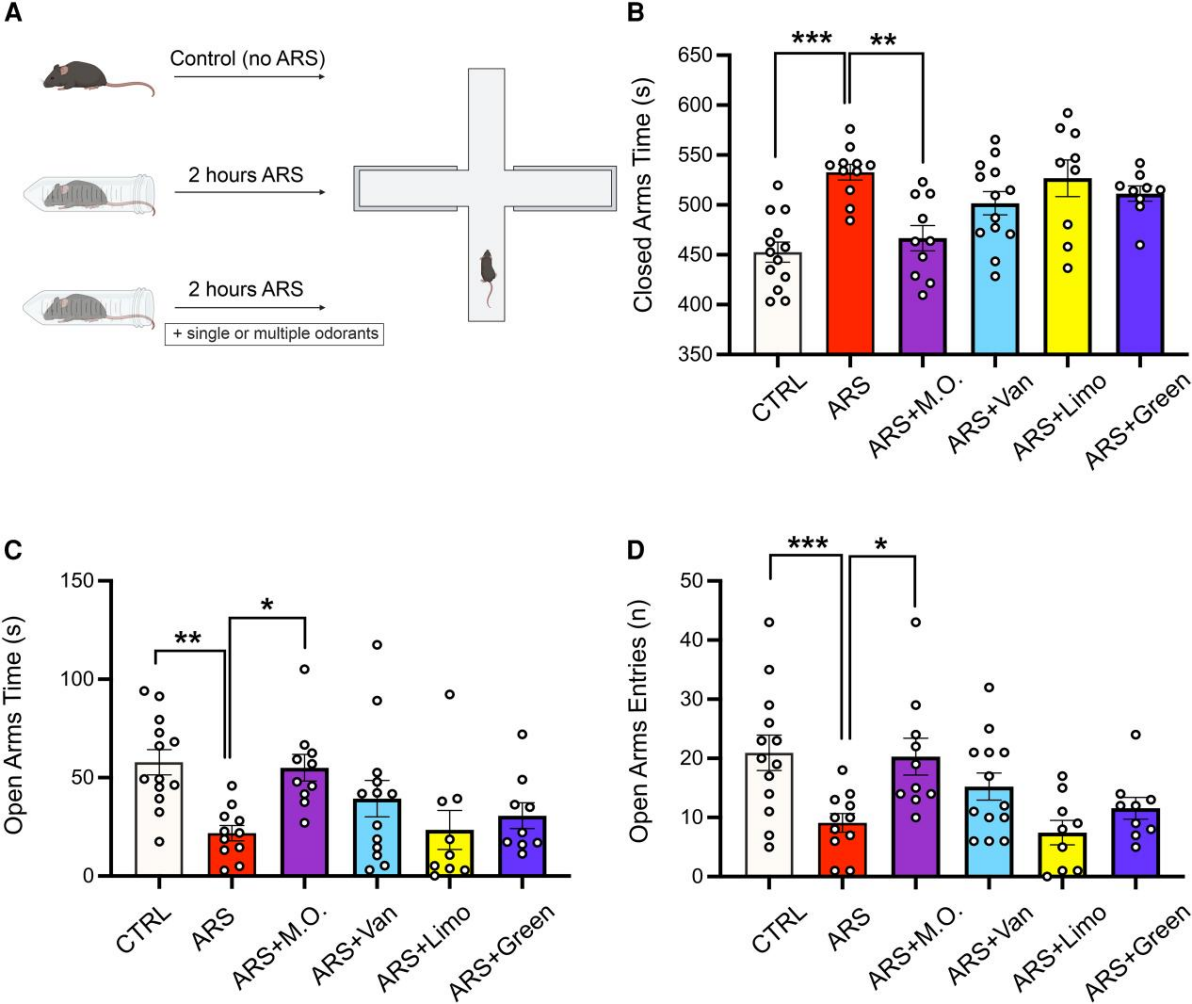
**Olfactory stimulation using multiple odorants prevents stress induced anxiety.** (**A**) Schematic representations of the experimental protocol. (**B**, **C** and **D**) Evaluation of the behavioral profile of animals exposed to the acute restraint stress (ARS) protocol in the presence of either a single or multiple odorants (ARS-M.O.). ARS-M.O. exposed animals were the only group showing a significant reduction of time spent into closed arms (**B**) and a significant increase in time spent and entries into open arms **C** and **D**; CTRL n = 13; ARS n = 11; ARS + M.O. n = 10; ARS + Vanillin n = 13; ARS + Limonene n = 9; ARS + Green Odor n = 9). All data are reported as mean ± s.e.m. Dots represent the number of samples (studied animals). Statistics by One-Way ANOVA followed by post-hoc Holm-Sidak (**B**, **D**), One-Way ANOVA on Ranks, post-hoc Dunn’s methods (**C**); *P < 0.05; **P < 0.01; ***P < 0.001.

Once tested the efficacy of our stress paradigm, we investigated the effects of olfactory stimulation on ARS-induced behavioral alterations. Specifically, animals were exposed to three different odorants, i.e. vanillin, limonene and green odor to compare the effects of a single versus multiple odorant stimulation (Fig. 2A). Exposure to a single odorant did not halt the behavioral alterations induced by ARS procedure in terms of time spent in the closed and open arms (closed arms time: CTRL 452.6 ± 10.1 s; ARS 532.7 ± 7.8 s; ARS-Vanillin 501.6 ± 11.7 s; ARS-Limonene 526.7 ± 18.4 s; ARS-Green Odor 511.3 ± 7.6 s; One-way ANOVA, F_(5,64)_ = 7.948, *P* < 0.001; post-hoc Holm-Sidak method: CTRL versus ARS: *P* < 0.001; CTRL versus ARS-Limonene: *P* < 0.001; CTRL versus ARS-Green Odor: *P* =0.010; CTRL versus ARS-Vanillin: *P* = 0.019; open arms time: CTRL 57.8 ± 6.6 s; ARS 21.7 ± 4 s; ARS-Vanillin 39.3 ± 9.6 s; ARS-Limonene 23.4 ± 10.5 s; ARS-Green Odor 30.6 ± 6.9 s; ANOVA on Ranks, *P* < 0.001; post-hoc Dunn’s method: CTRL versus ARS: *P* = 0.010; CTRL versus ARS-Limonene *P* = 0.011), as well as the number of open arms entries (CTRL 21.0 ± 3.1; ARS 8.8 ± 1.3; ARS-Vanillin 15.2 ± 2.4; ARS-Limonene 8.3 ± 2.4; ARS-Green Odor 11.5 ± 2.2; One-way ANOVA, F_(5,64)_ = 5.197, *P* <0.001; post-hoc Holm-Sidak method: CTRL versus ARS: *P* = 0.005; CTRL versus ARS-Limonene: *P* = 0.011; Fig. 2C and D). However, stressed mice exposed to all odorants showed a significant decrease in time spent in closed arms (466.6 ± 13.4 s, *P* = 0.003 versus ARS), a significant increase in time spent in the open arms (55 ± 7.1 s, *P* = 0.038 versus ARS) as well as increased number of entries in the open arms (19.7 ± 3.5, *P* = 0.031 versus ARS) compared to ARS animals (Fig. 2B and C and D).

### Olfactory stimulation with multiple odorants halts the effects of ARS on recognition memory

**Figure 3:**
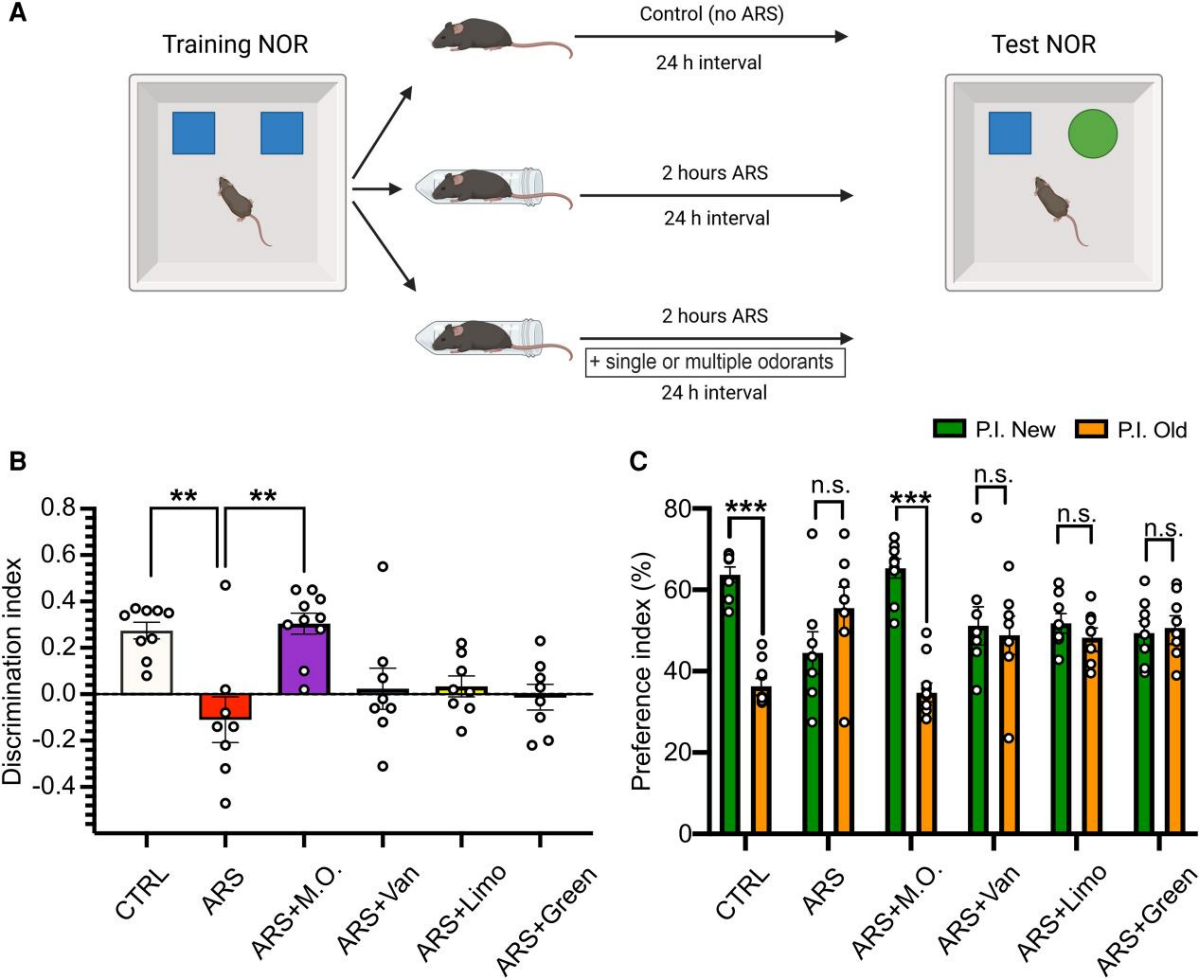
**Stress induced recognition memory deficits are prevented by exposure to multiple odorants.** (**A**) Schematic representations of the experimental protocol. (**B**) Analysis of the discrimination index of the test phase reveals a significant difference between CTRL and acute restraint stress (ARS) exposed animals and between ARS and ARS-M.O. exposed animals, suggesting functional recovery. (**C**) Preference index for the novel (P.I. New, green) and old (P.I. Old, orange) objects. Only CTRL and ARS-M.O. animals spent a significantly higher proportion of time exploring the novel compared to the already known object (CTRL n = 9; ARS n = 8; ARS + M.O. n = 10; ARS + Vanillin n = 8; ARS + Limonene n = 8; ARS + Green Odor n = 8). All data are reported as mean ± s.e.m. Dots represent the number of samples (studied animals). One-Way ANOVA on Ranks followed by post-hoc Dunn’s method (**B**), Student’s *t* test (**C**); **P < 0.01; ***P < 0.001.

It has been reported that stressful stimuli also affect recognition memory.^21^ Thus, we investigated memory performance after exposure to ARS protocol associated with olfactory stimulation using either a single or multiple odorants. Animals were exposed to the ARS procedure during the consolidation phase of the NOR training, right after the training phase, and they were tested 24 h later (Fig. 3A). ARS-exposed mice showed memory impairment with a significant reduction of the D.I. compared to control animals, and the same pattern was observed in all ARS mice exposed to a single odorant (D.I.: CTRL 0.27 ± 0.04; ARS −0.11 ± 0.1; ARS-Vanillin 0.02 ± 0.09; ARS-Limonene 0.04 ± 0.05; ARS-Green Odor 0.01 ± 0.06; ANOVA on Ranks, *P* < 0.001; post-hoc Dunn’s Method: CTRL versus ARS: *P* = 0.009, Fig. 3B). Moreover, as shown in Fig. 3C, the P.I. for the novel and old object did not differ in ARS-exposed animals among vehicle- and each single odorant-treated mice (P.I. New versus P.I. Old, respectively: CTRL 63.7 ± 1.9% versus 36.3 ± 1.9%; ARS 44.5 ± 5.2% versus 55.5 ± 5.2%; ARS-Vanillin 51.1 ± 4.7% versus 48.8 ± 4.7%; ARS-Limonene 51.7 ± 2.4% versus 48.2 ± 2.4%; ARS-Green Odor 50.6 ± 2.9% versus 50.6 ± 2.9%; Student’s *t*-test, P.I. novel versus P.I. old: CTRL, t = 10.785, *P* < 0.001; ARS, t = 1.59, *P* = 0.133; Vanillin, t = 0.368, *P* = 0.718; Limonene, t = 1.112, *P* = 0.284; Green Odor, t = 0.323, *P* = 0.750, Fig. 3C). Of note, animals exposed to ARS along with multiple odorants showed a full recovery in both the preference and discrimination indexes (D.I. ARS-M.O. 0.31 ± 0.05 post-hoc Dunn’s Method: ARS versus ARS-M.O.: *P* = 0.003; P.I. New versus P.I. Old: ARS-M.O. 65.3 ± 2.3% versus 34.7 ± 2.3%; Student’s *t*-test, ARS-M.O., t = 9.673, *P* < 0.001, Fig. 3B and C). Finally, total exploration was not altered by either olfactory stimulation or stress exposure, with the only significant difference observed between Limonene- and Green Odor ARS-exposed animals (Table 1, One-way ANOVA, F_(5,45)_ = 2.741, *P* = 0.030; post-hoc Holm-Sidak method: Limonene versus Green Odor: *P* = 0.034).

**Table 1:** Exploration times in the test phase of the NOR and OPR tests

	CTRL	ARS	ARS + M.O.	ARS + Vanillin	ARS + Limonene	ARS + Green Odor
NOR	19.3 ± 1.7 s	16.1 ± 1.4 s	15.0 ± 2 s	15.2 ± 2.9 s	12.3 ± 1.3 s	22.3 ± 3.1 s
OPR	15.1 ± 1.7 s	10.6 ± 1.1 s	14.4 ± 2 s	11.6 ± 1.3 s	14.3 ± 2.4 s	14.9 ± 1 s

### Olfactory stimulation with multiple odorants halts the effects of ARS on spatial memory consolidation

**Figure 4:**
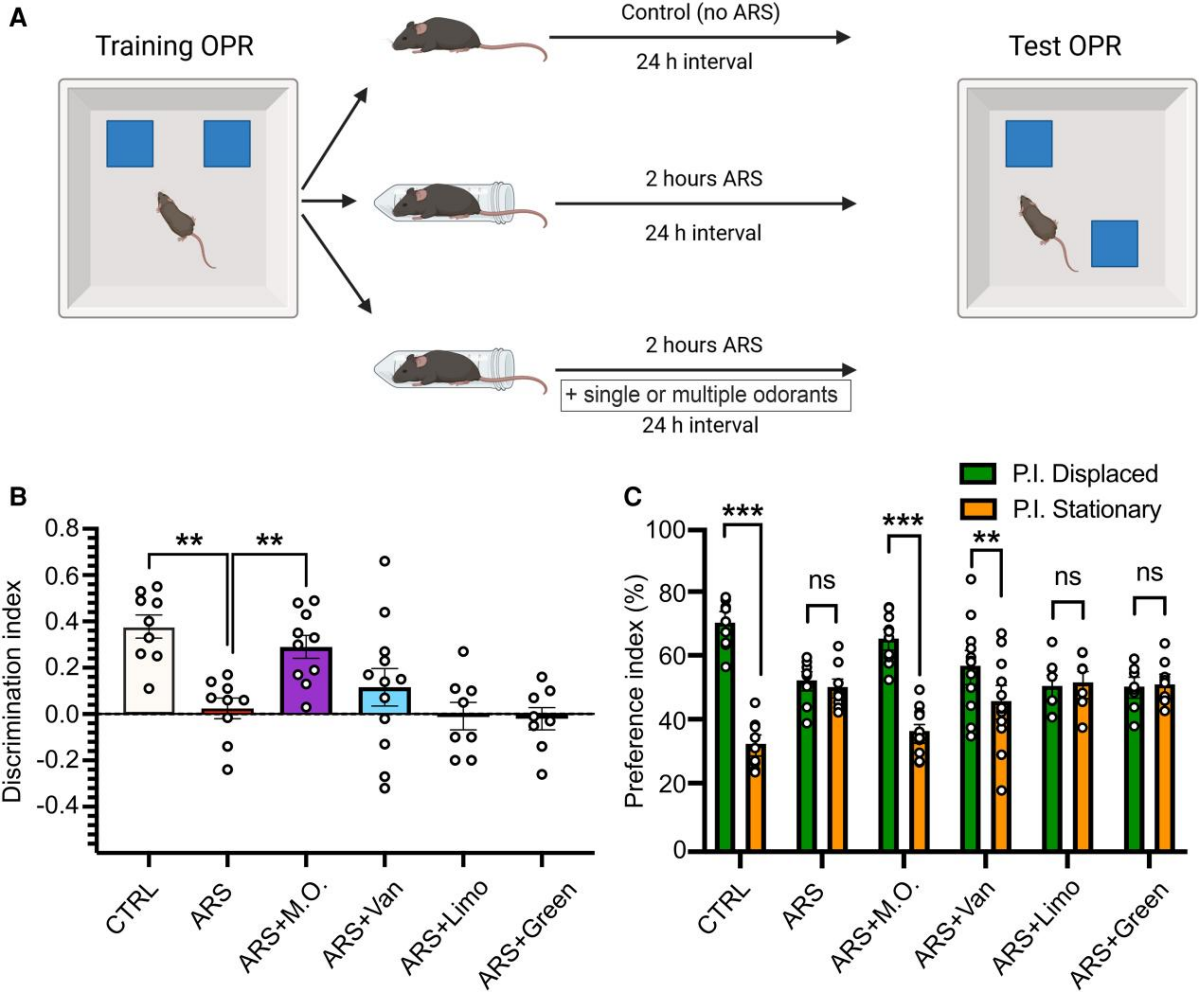
**Stress induced recognition memory deficits are prevented by exposure to multiple odorants.** (**A**) Schematic representations of the experimental protocol. **(B**) CTRL and acute restraint stress (ARS) M.O. exposed animals had a significantly higher discrimination index compared to ARS-exposed exposed mice. (**C**) Analysis of the P.I.s for the displaced (P.I. Displaced, green) and stationary (P.I. Stationary, orange) objects revealed that CTRL, ARS-M.O. and ARS-Vanillin mice spent a significantly higher proportion of time exploring the displaced object (CTRL n = 9; ARS n = 9; ARS + M.O. n = 11; ARS + Vanillin n = 12; ARS + Limonene n = 8; ARS + Green Odor n = 8). All data are reported as mean ± s.e.m. Dots represent the number of samples (studied animals). One-Way ANOVA followed by post-hoc Holm-Sidak’s method (**B**), Student’s *t* test (**C**); **P < 0.01; ***P < 0.001.

Next, we employed the same paradigm to evaluate the impact of olfactory stimulation on spatial memory consolidation under stressful conditions. Specifically, animals were subjected to ARS and olfactory stimulation since the end of OPR training phase for 2 h, which is considered the time for the consolidation process,^28^ and they were tested 24 h later (Fig. 4A). Again, ARS-exposed animals, as well as ARS-Limonene- and ARS-Green Odor exposed animals, showed spatial memory impairment, as revealed by a significant reduction of the discrimination index (DI) compared to CTRL animals (D.I.: CTRL 0.38 ± 0.05; ARS 0.02 ± 0.5; ARS-Limonene −0.01 ± 0.06; ARS-Green Odor −0.02 ± 0.05; One-way ANOVA, F_(5,55)_ = 7.189, *P* < 0.001; post-hoc Holm-Sidak method: CTRL versus ARS *P* = 0.007; CTRL versus ARS-Limonene *P* = 0.003; CTRL versus ARS-Green Odor *P* = 0.004, Fig. 4B), further confirmed by the analysis of the P.I. for the displaced and stationary objects (P.I. Displaced versus P.I. Stationary, respectively: CTRL 68.9 ± 2.6% versus 31.1 ± 2.6%; ARS 51.2 ± 2.4% versus 48.7 ± 2.4%; ARS-Limonene 49.5 ± 3.1% versus 50.4 ± 3.1%; ARS-Green Odor 49.0 ± 2.5% versus 51.0 ± 2.5%; Student’s *t*-test, P.I. Displaced versus P.I. Stationary: CTRL, t = 10.786, *P* < 0.001; ARS, t = 0.762, *P* = 0.456; ARS-Limonene, t = 0.224, *P* = 0.825; ARS-Green Odor, t = 0.57, *P* = 0.577, Fig. 4C). Of note, when analyzing the P.I. for the displaced and stationary objects, but not the D.I., ARS-Vanillin exposed animals spent significantly higher time exploring the displaced object compared to the stationary object, suggesting memory recovery (D.I. ARS-Vanillin 0.16 ± 0.08; P.I. Displaced versus Stationary: ARS-Vanillin 57.7 ± 4% versus 42.2 ± 4%; t = 2.851, *P* = 0.009). In agreement with the results of the NOR test, ARS-M.O. animals showed an increased preference for the displaced object compared to the stationary one, which was not observed in ARS animals, as well as a significant increase in the D.I. compared to ARS-exposed animals (D.I. ARS-M.O. 0.29 ± 0.05, ARS versus ARS-M.O. *P* = 0.027; P.I. Displaced versus Stationary: ARS-M.O. 64.4 ± 2.5% versus 35.5 ± 2.5%; t = 8.413, *P* < 0.001). Finally, total exploration was not altered by olfactory stimulation nor by stress exposure (Table 1, One-way ANOVA, F_(5,55)_ = 1.480, *P* = 0.214).

### Olfactory stimulation counteracts the unpredictable chronic stress-related behavioral alterations

**Figure 5:**
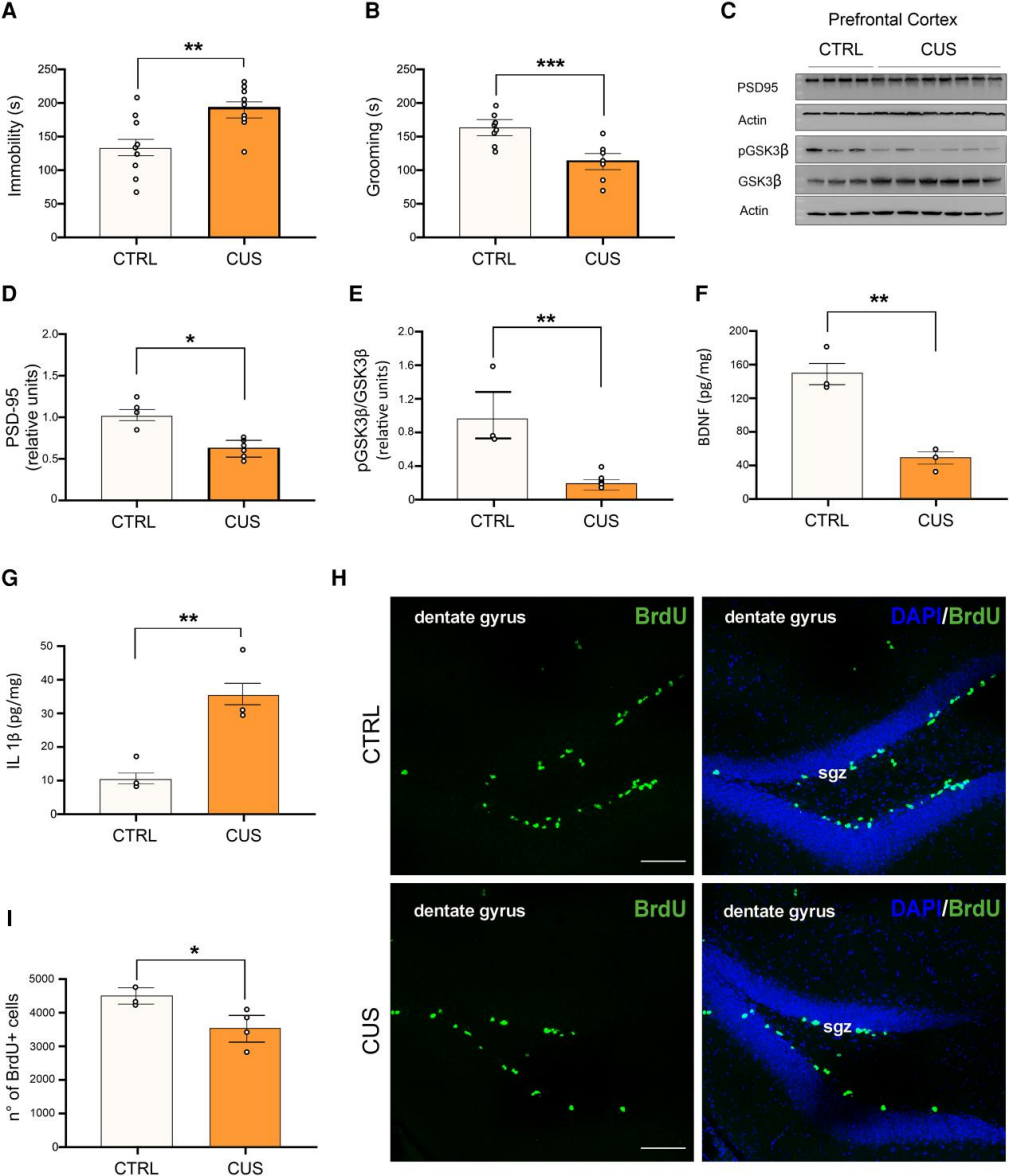
**CUS-exposed animals show behavioral, molecular, and biochemical hallmarks of chronic stress response.** Animals were subjected to 6 weeks of chronic unpredictable stress (CUS). (**A)** and (**B**) At the end of the 6 weeks period, CUS-exposed animals showed a significant decrease in the time spent performing self-grooming in the SST (**A**) and a significant increase in time of immobility in the FST (B, n = 8 for both groups). (**C**, **D** and **E**) Representative blots showing alterations in expression and phosphorylation of different molecular substrates. CUS-exposed animals show reduced PSD-95 levels (n = 4 control mice, n = 8 CUS mice), as well as decreased GSK3β inhibitory phosphorylation on Ser9 (n = 3 control mice, n = 6 CUS mice). (**G**) IL 1β and BDNF levels after CUS-exposure. Depressed mice show an increase in IL 1β in the PFC as well as a decrease in BDNF levels (n = 3 control mice, n = 3 CUS mice). (**H** and **I**) Representative images and quantification of the total number of proliferating neural stem cells in the hippocampus of both CTRL and CUS-exposed animals (see ‘Materials and Methods’ section for the quantification method; n = 3 control mice, n = 4 CUS mice; Scale bar: 100 µm); sgz: subgranular zone. Dots represent the number of samples (studied animals). Student’s t test was performed for all comparisons. All data are reported as mean ± s.e.m. *P < 0.05; **P < 0.01, ***P < 0.001. See supplementary materials for uncropped blots.

**Figure 6:**
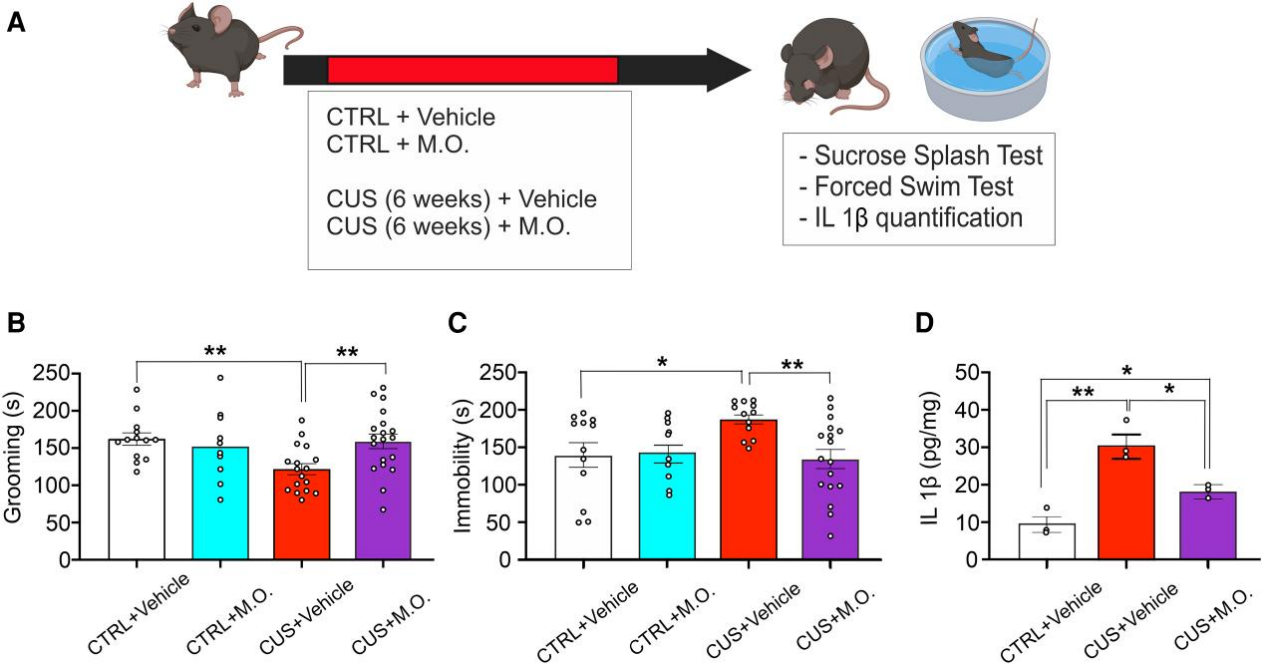
**Olfactory stimulation prevents the development of a depressive phenotype in CUS-exposed mice.** (**A**) Schematic representation of the experimental protocol. Animals were divided into four groups: 1) control unstressed mice exposed to vehicle stimulation; 2) control unstressed mice exposed to multiple odorants stimulation; 3) CUS-exposed mice treated with vehicle stimulation; 4) CUS-exposed mice treated with multiple odorants stimulation. (**B**) Time spent in the grooming during the sucrose splash test for CTRL + vehicle, CTRL + M.O., CUS + vehicle and CUS + M.O. groups (CTRL + vehicle n = 13; CTRL + M.O. n = 10; CUS + vehicle n = 17; CUS + M.O. n = 19). (**C**) Time of immobility during the forced swim test for CTRL, CUS + Vehicle and CUS + M.O. groups (CTRL + vehicle n = 12; CTRL + M.O. n = 10; CUS + vehicle n = 16; CUS + M.O. n = 17). (**D**) IL 1β levels in the PFC of CTRL + vehicle, CUS + vehicle and CUS + M.O. stimulation (n = 3 each group). Dots represent the number of samples (studied animals). Two-way ANOVA followed by post-hoc Holm-Sidak method (B and (**C**); One-way ANOVA followed by post-hoc Holm-Sidak method (**D**). All data are reported as mean ± s.e.m. *P < 0.05; **P < 0.01.

Next, we tested whether the combination of the studied odorants prevented mood alterations induced by a chronic unpredictable stress paradigm to evaluate a possible translational efficacy of said mixture against the long-term consequences of stress. First, we characterized our CUS model at behavioral, molecular and cellular levels. Six-weeks of CUS protocol induced a significant increase in the immobility time of CUS-exposed mice compared to controls in the forced swimming test (192 ± 11 s versus 134 ± 16 s respectively, Student’s t test, t = 3.158, *P* = 0.006, Fig. 5A), as well as a significant reduction of time spent performing self-grooming in the sucrose splash test (114 ± 8 s versus 162 ± 7 s, Student’s t test, t = 4.439, *P* < 0.001, Fig. 5B). Both behavioral changes did not depend on altered locomotor activity, as no difference between total distance traveled was recorded in the open field test (Supplementary Fig. 1). Animals were then sacrificed for molecular and immunofluorescence analyses. CUS-treated animals showed in the PFC several characteristic molecular alterations of chronic stress exposure^22,29^ including a significant reduction of PSD-95 levels (38% reduction in CUS-exposed animals compared to control mice, Student’s *t* test, t = 3.240, *P* = 0.010, Fig. 5C and D) and a decrease of GSK3β serine 9 phosphorylation (81% reduction in CUS-exposed mice compared to control animals, Student’s t test, t = 3.831, *P* = 0.006, Fig. 5C and E). A significant reduction in BDNF levels as well as a significant increase in IL 1β concentration were also observed in the PFC of CUS-exposed mice (BDNF: 50.7 ± 7.1 pg/mg versus 150.7 ± 13.2 pg/mg, Student’s *t* test, t = 5.771, *P* = 0.004, Fig. 5F; IL 1β: 36 ± 5.7 pg/mg versus 10 ± 1.8 pg/mg, Student’s *t* test, t = 3.782, *P* = 0.019, Fig. 5G). Furthermore, analysis of CUS hippocampi also revealed a reduction of neural stem cells proliferation compared to control mice (3601 ± 441 versus 4579 ± 225 BrdU positive cells, Student’s t test, t = 2.711, *P* = 0.042, Fig. 5H and I). Subsequently, we evaluated whether the multiple odorants stimulation rescued behavioral alterations in CUS-exposed animals. In the sucrose splash test, two-way ANOVA revealed no significant effect of CUS- or M.O.-exposure (Fig. 6B). Instead, a significant interaction CUS × M.O. was observed (Two-way ANOVA, F = 5.552, *P* = 0.022). Post-hoc tests revealed that mice exposed to both CUS and vehicle sprays had a significant reduction of their self-grooming activity compared to control animals as well as compared to animals exposed to both CUS and olfactory stimulation, which recovered to the level of control animals (CTRL + vehicle: 162.1 ± 8.6 s, CTRL + M.O.: 151.5 ± 15.6 s, CUS + vehicle: 121.7 ± 7.7 s, CUS + M.O.: 159 ± 10.1 s; post-hoc Holm-Sidak method: CTRL versus CUS-vehicle: *P* = 0.005; CUS-vehicle versus CUS-M.O.: *P* = 0.005). Similar results were obtained in the forced swim test, where an effect of the interaction between stress and M.O. was observed (Two-way ANOVA, F = 4.635, *P* = 0.036), as immobility time significantly increased in vehicle stimulated-CUS animals compared to both control and olfactory stimulated-CUS animals (CTRL + vehicle: 141.1 ± 17.2 s, CTRL + M.O.: 143.2 ± 15.9 s, CUS + vehicle: 188.7 ± 6.2 s, CUS + M.O.: 135.8 ± 13.2 s, post-hoc Dunn’s Method: CTRL versus CUS-vehicle: *P* = 0.010; CUS-vehicle versus CUS-M.O.: *P* = 0.002, Fig. 5C). No differences were observed between control-vehicle and control-M.O. mice in both tests. Finally, we investigated if the M.O. stimulation also reverted one of the molecular changes potentially involved in the development of CUS-induced depressed phenotype. Indeed, IL 1β levels in the PFC were significantly reduced by M.O. exposure compared to CUS-vehicle animals, although they still were significantly higher compared to control animals (One-way ANOVA, F_(2,8)_ = 21.189; *P* = 0.002; CTRL + vehicle: 10 ± 1.7 pg/mg; CUS + vehicle: 30.6 ± 2.9 pg/mg; CUS + M.O.: 18.4 ± 0.8 pg/mg; post-hoc Holm-Sidak method: CTRL + vehicle versus CUS + vehicle: *P* = 0.002; CUS-vehicle versus CUS-M.O.: *P* = 0.018; CTRL + vehicle versus CUS + M.O.: *P* = 0.038, Fig. 6D).

## Discussion

Several reports have shown that vanillin, limonene, or green odor molecules, when either inhaled or orally/intraperitoneally administered, induce beneficial effects on brain function. It has been reported that green odor exposure can increase the time spent in open arms of the EPM, and both dopamine and serotonin levels in the brain in non-stressed animals.^30-32^ It can also reduce hypothalamic parvalbumin positive neurons activity and adrenocorticotropic hormone release^33,34^ as well as inducing a different activity pattern in limbic system regions.^35^ Limonene inhalation, both as single molecule and in essential oils obtained from bergamot or orange, has been reported as well to reduce anxious behaviors under no stress conditions^36-38^ or to reduce depressive behaviors in chronic stress mouse model.^17^ Finally, vanillin has been shown to impact mood alterations in a model of depression^20^ but it failed to show an anxiolytic effect under no stress condition in different tests, such as the EPM, the open field test and the FST.^39^ In this work, we report that olfactory stimulation with a combination of multiple odorants applied during stressful conditions counteracts the detrimental outcome of stress exposure on behavior, whereas single odorant stimulation is either ineffective or scarcely effective in an experimental model of acute stress. Furthermore, we show that the selected mix of odorants prevents the development of depressive symptoms in a mouse model of chronic stress.

In the ARS model, we observed biochemical, molecular and behavioral alterations that are typical of the acute stress response. Therefore, we sought to determine which odorant and/or whether a combination of the three could better halt stress-induced alterations. In the EPM, the stimulation with multiple odorants was able to induce a functional recovery on the different parameters recorded, which was not obtained when using any of the single odorants. In the NOR and OPR tests, stimulation with multiple odorants induced a full recovery of recognition and spatial memory. Of note, vanillin was the only odorant that was able to induce a significant recovery of function in the spatial memory test. Overall, exploratory activity of the objects was reduced by the stress paradigm and, in some cases, such as green odor and limonene stimulation in the NOR test, the single odorants were able to induce an increase in the exploratory function. In the chronic stress paradigm, we observed behavioral, cellular and molecular alterations previously shown to be associated with a depressive phenotype. When animals were exposed to all the odorants, they showed no depressive behaviors in both the sucrose splash test and in the forced swim test, thus confirming that the selected mixture also holds a prophylactic efficacy not only against the short-term consequences of acute stress, but also toward long-term stress-induced alterations. Furthermore, a representative stress-induced molecular change such as IL 1β levels was reverted in the PFC of CUS-M.O. exposed animals compared to the CUS-vehicle group, therefore pointing toward a potential role of odorants stimulation in counteracting stress-dependent alterations including inflammatory signaling.

Our data add a novel layer of knowledge about the effects of olfactory stimulation on limbic system activity and stress-dependent behavioral alterations. Compared to previous works, some discrepancies may arise and three important distinctions must be defined in the context of literature reports: i) whether the stress is present or not; ii) stress duration and iii) odorant concentration. Some works have evaluated the effect of olfactory stimulation under basal condition (i.e. no stress), reporting a positive effect for single odorants on anxious behavior which doesn’t automatically translates into a single odorant being beneficial in halting stress-induced anxiety. Stress duration may also represent an important variable in understanding how olfactory responses shape stress effects. For example, Lee *et al*. has demonstrated that exposure to a single odorant, such as 2-phenylethanol or hinokitiol, can reduce behavioral alterations induced by different types of stress, through the regulation of corticotropin releasing hormone neurons in the hypothalamus.^10^ However, the restraint stress protocol they used lasted 10 min, which may induce a milder response compared to a 2 h stress paradigm which could not allow for a recovery through single odorant exposure. Finally, odorant concentration also plays a role. For example, it has been reported that the effect of bergamot essential oil on anxious behavior can be modulated by the odorant concentration, with more behavioral parameters being modified by higher concentrations of the volatile compounds.^37^ Again, other reports have shown a dose-dependent efficacy of limonene containing mixtures on behavioral parameters.^36^ In this work, we employed low concentrations, which were calculated based on our previous studies^40^ to evaluate if the combination of different odorants *per se* could still produce an effect on stress response.

We do not claim that the specific mixture of odorants we employed is the only one effective in counteracting the effects of acute and chronic stress. It is possible that other combinations of multiple odorants may exert similar additive or synergistic actions, resulting in even a greater effect. For example, measuring the effect size of our olfactory stimulation between CUS-vehicle and CUS-M.O. mice in the FST reveals a Cohen’s *d* value of 1.08. A recent meta-analysis on the effect size of classical antidepressant drugs evaluated in chronic stress models in the FST revealed an average effect size of 2.44.^41^ While we did not add a positive control group (i.e. a group treated with antidepressants) for a more accurate evaluation of the different effect sizes, a comparison with the above cited results suggests that the proposed mixture can still be improved to match standard antidepressant therapies, by adding other odorants in the stimulation protocol or employing other types of odorants. Indeed, stimulation by different odorants may overcome the adaptation of olfactory receptors thus triggering a significantly higher sensory input through the olfactory nerve. Olfactory habituation is a well-known phenomenon in which an odorant is not perceived after prolonged exposure, due to the desensitization of its receptors.^42^ The stimulation with a single odorant may therefore induce the deactivation of the specific receptor, leading to reduced olfactory stimulation. On the other hand, alternatively using multiple odorants could maintain a higher stimulation level compared to single stimulation. Nonetheless, each odorant may exert a specific action thus inducing a different physiological effect. For instance, limonene inhalation, but not *Melaleuca alternifolia* essential oils, have been shown to impact on anxious behavioral profiles.^38^ Here we report that vanillin prevents the detrimental effect of stress on spatial memory consolidation. This effect may be explained by vanillin affinity for the transient receptor potential vanilloid 1,^43^ which is involved in pain perception and in memory storage and is thoroughly expressed in the hippocampus.^44,45^ Indeed, it is possible that odor stimulation exerts its action either by stimulation of the olfactory pathway leading to the regulation of limbic regions activity or by absorption of volatile molecules in the nasal cavity through the nasal mucosa, which can then enter the blood flow to reach and bind specific receptors.

Neuroinflammation is one of the multiple pathways involved in the etiopathogenesis of stress-induced mood disorders, as also shown by our characterization of the CUS-model. Among the multiple pro-inflammatory cytokines, IL 1β is at the crossroad of different signaling cascades regulating learning and memory, neurogenesis, glial reactivity, neuronal excitability etc., both in physiological and pathological conditions. A recent work has demonstrated that lavender essential oils can reduce the levels of multiple pro-inflammatory cytokines, such as IL 1β and tumor necrosis factor α, in the central amygdala of a mouse model of visceral pain.^46^ These data are in agreement with our findings, as we also observed a reduction of IL 1β in CUS-M.O. exposed mice compared to CUS-vehicle mice, although in a different brain area. Given its importance, we focused our attention on IL 1β but we cannot exclude the role of other pro-inflammatory molecules nor the role of other molecular pathways mediating the effects of M.O. stimulation. Indeed, M.O. stimulation induces only a partial recovery of PFC IL 1β levels, being significantly lower compared to CUS-vehicle animals yet higher than control animals, thus suggesting that M.O. stimulation involves other mechanisms beyond IL 1β reduction.

Collectively, our data suggest that, even in acute conditions, olfactory stimulation using a mixture of different odorants can have a prophylactic action on the detrimental consequences of an acute stressor and show that, even a simple mixture of three odorants, can prevent depressive behavior induced by chronic stress. Furthermore, our results point toward the existence of a tight connection between olfactory function and the regulation of limbic inflammatory signaling. Our data underline the possibility that the use different mixtures of odorants may be more effective in treating stress-induced conditions than exposure to a single odorant. Further experiments will be necessary to obtain a thorough insight on the mechanisms of action of olfactory stimulation, to better characterize which brain areas are more affected by such treatments and how they can be exploited in clinical settings.

## Supplementary Material

fcae390_Supplementary_Data

## Data Availability

Data are available on request to the corresponding author.
