# Supplementary material for: Olfactory stimulation with multiple odorants prevents stress-induced cognitive and psychological alterations
Source: Brain Commun. 2024 Nov 5;6(6):fcae390. doi: 10.1093/braincomms/fcae390 (PMC11574619; doi:10.1093/braincomms/fcae390)
Supplement: fcae390_Supplementary_Data [file fcae390_supplementary_data.pdf]

# Supplementary Materials

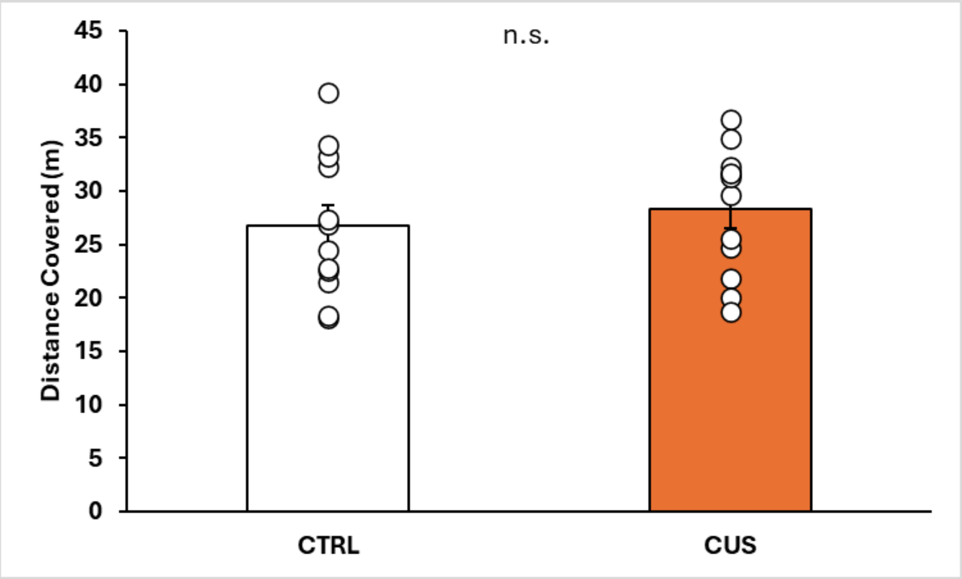


**Supplementary Figure 1. Evaluation of locomotor activity in control mice and CUS-exposed mice.** No differences were observed in locomotor activity between control mice and CUS-exposed mice (n=12 each condition). Student's *t* test. Each dot represents one animal sample. All data are reported as mean ± s.e.m. n.s. not significant.

**Uncropped gels: samples beyond the white squares are unrelated to the experiment.**

**Figure 1**

WB ventral hippocampus

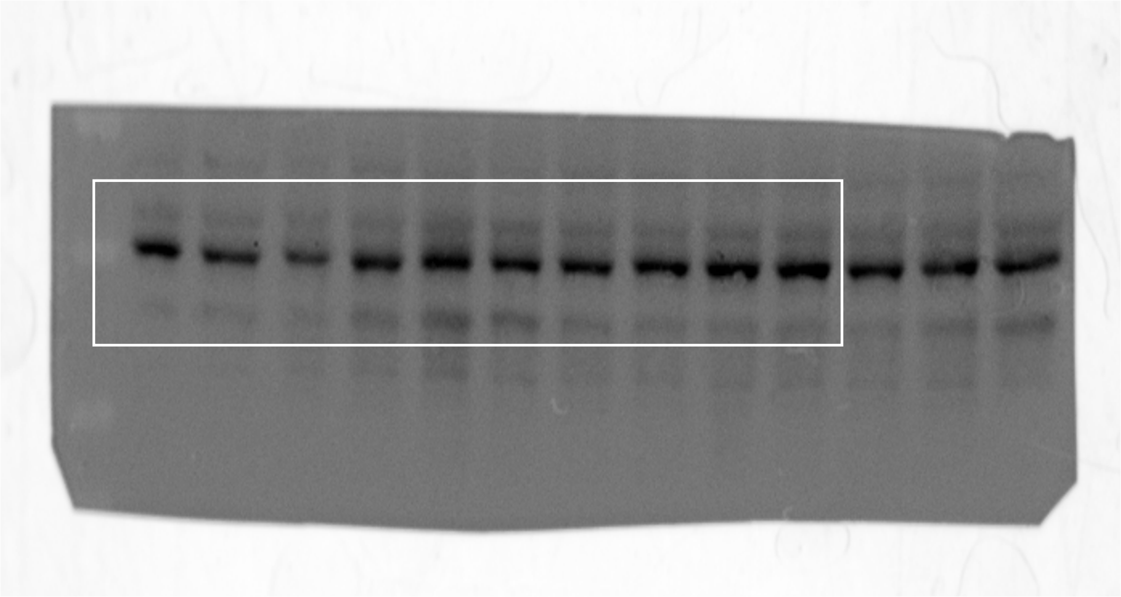

pERK

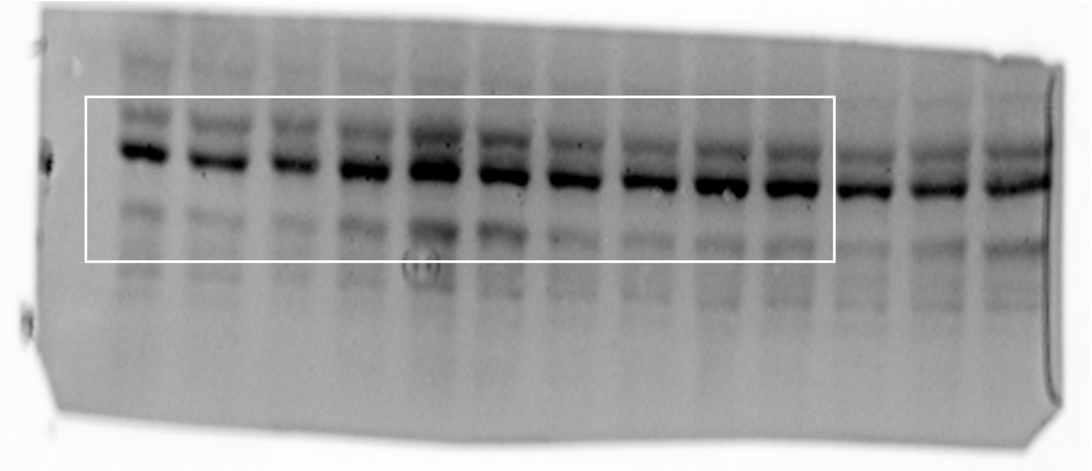

ERK

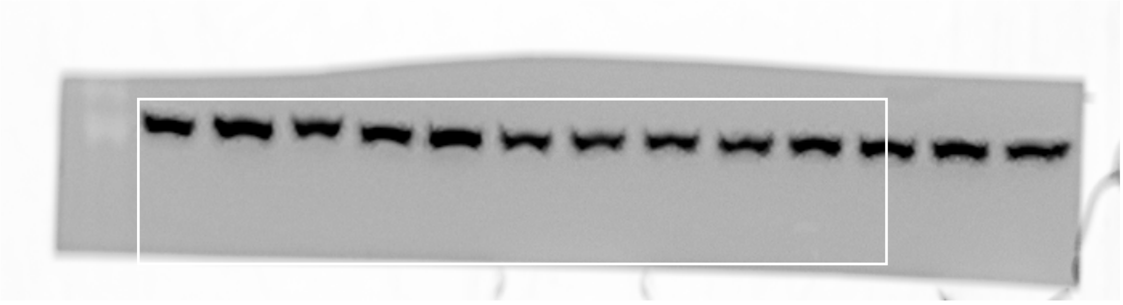

Hsp90

WB prefrontal cortex

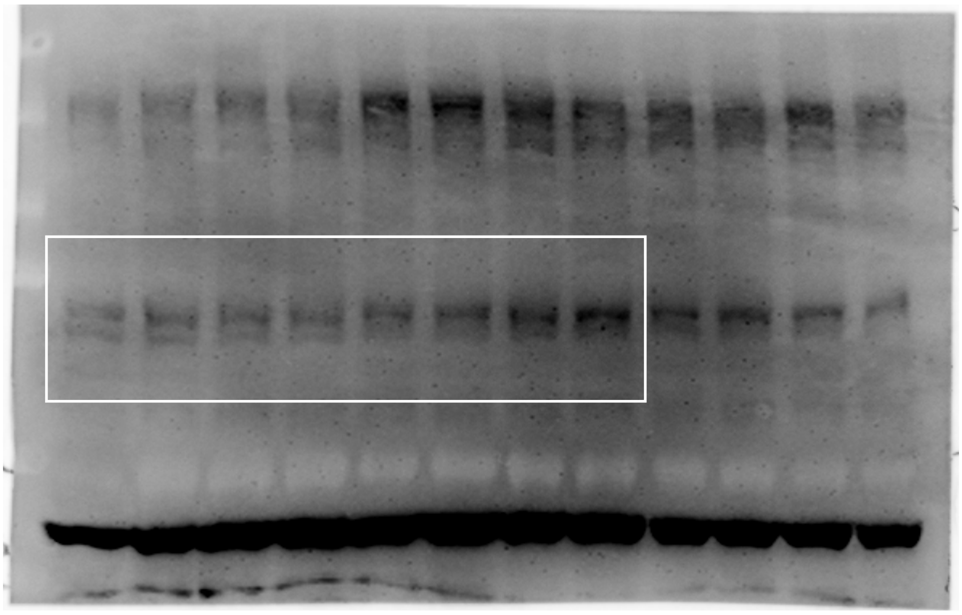

pNf-kB

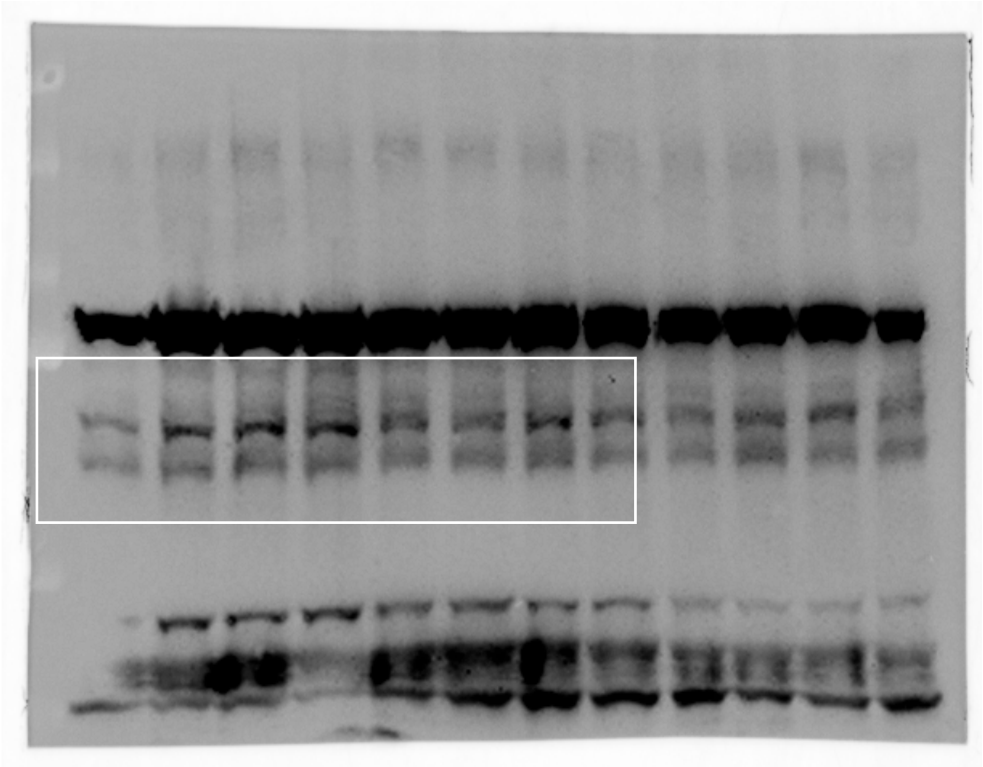

Nf-kB

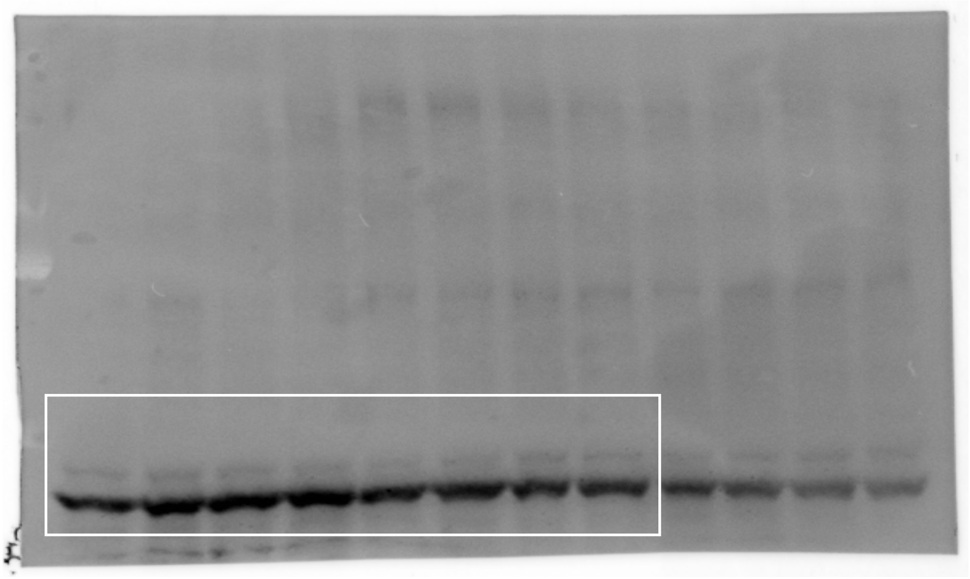

pERK

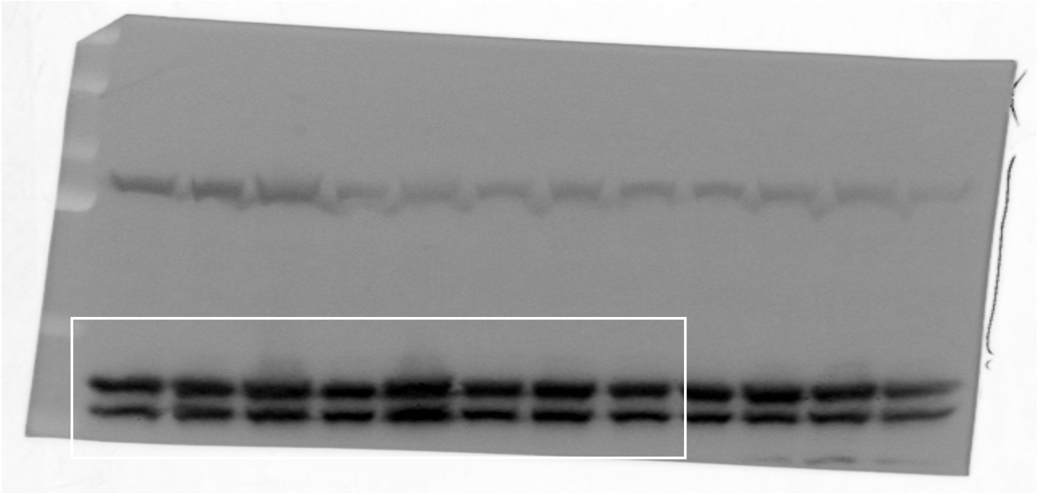

ERK

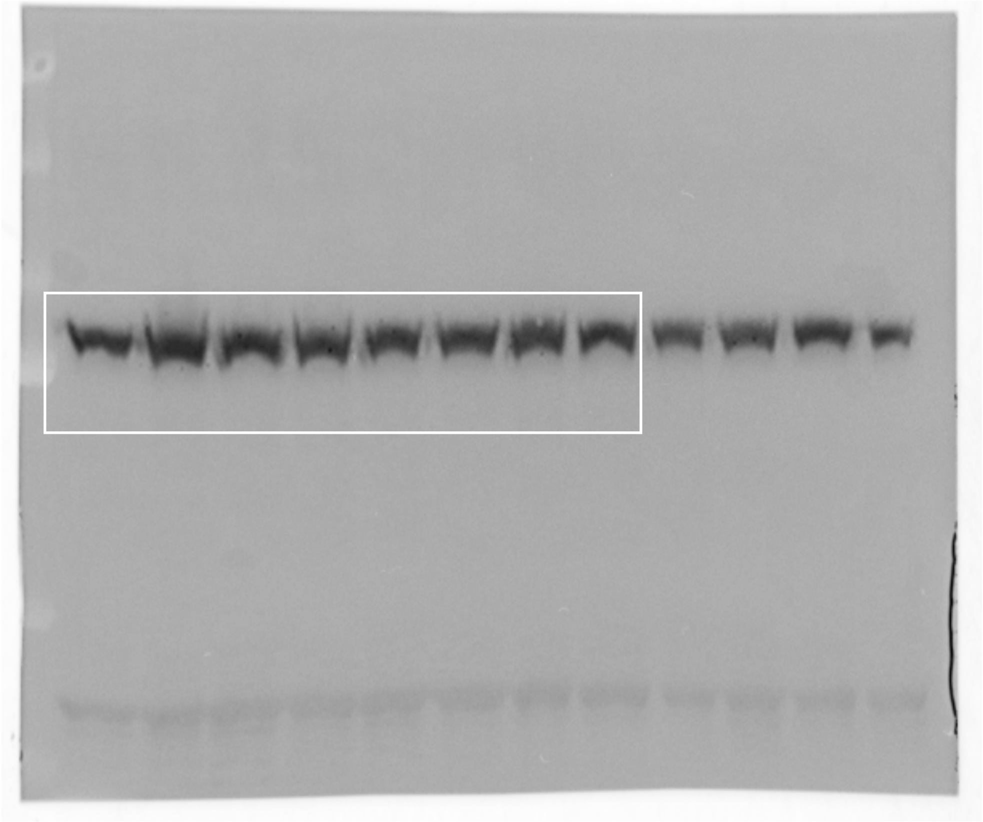

Hsp90

**Figure 5**

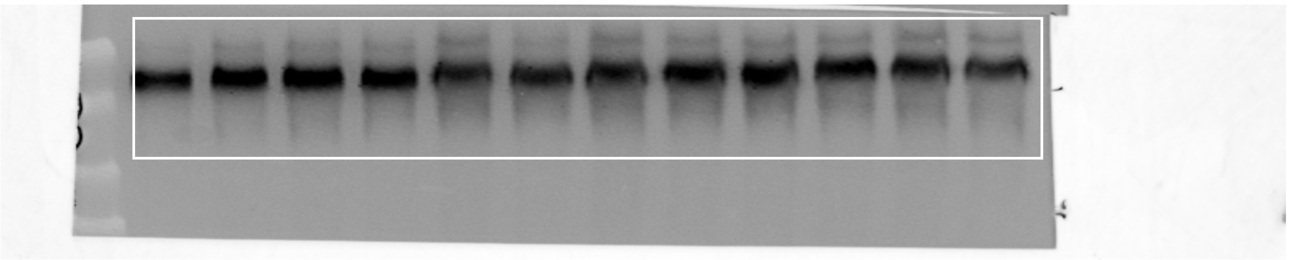

PSD-95

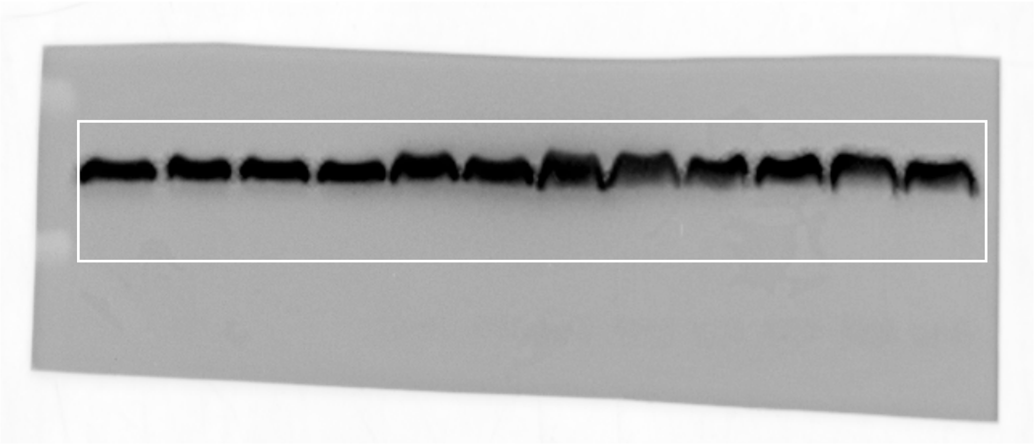

Actin

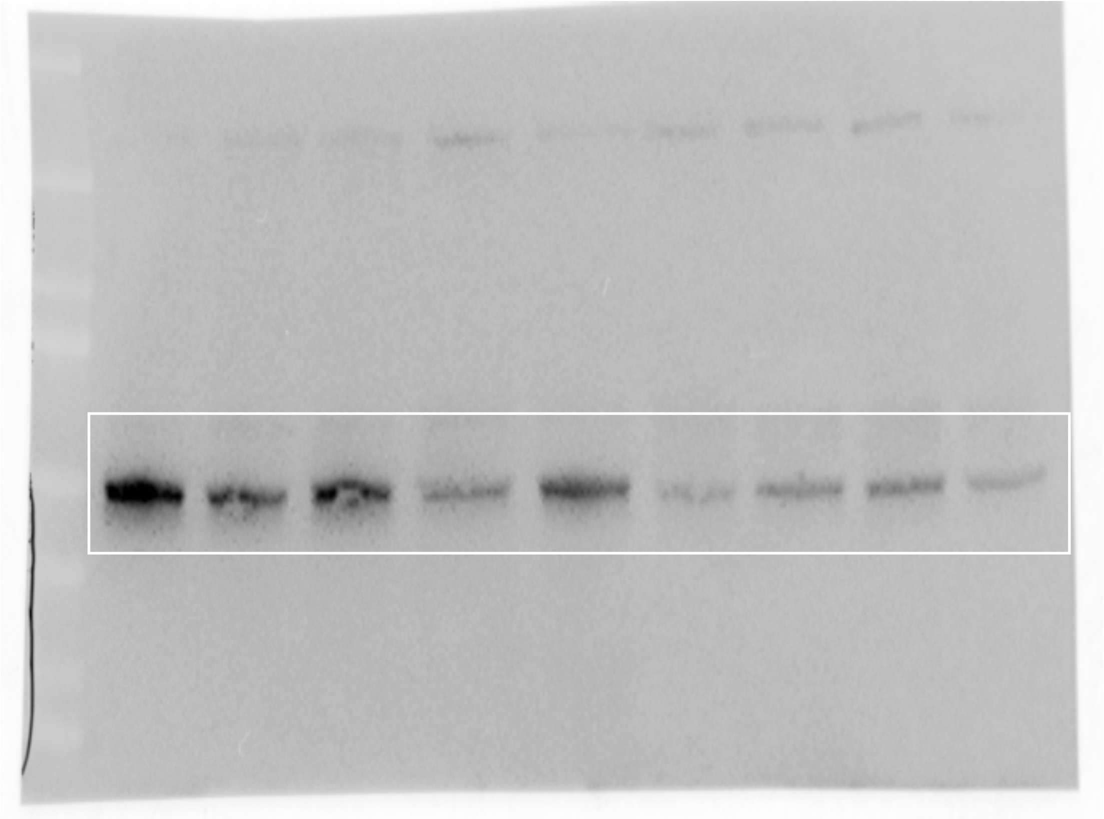

pGSK3β

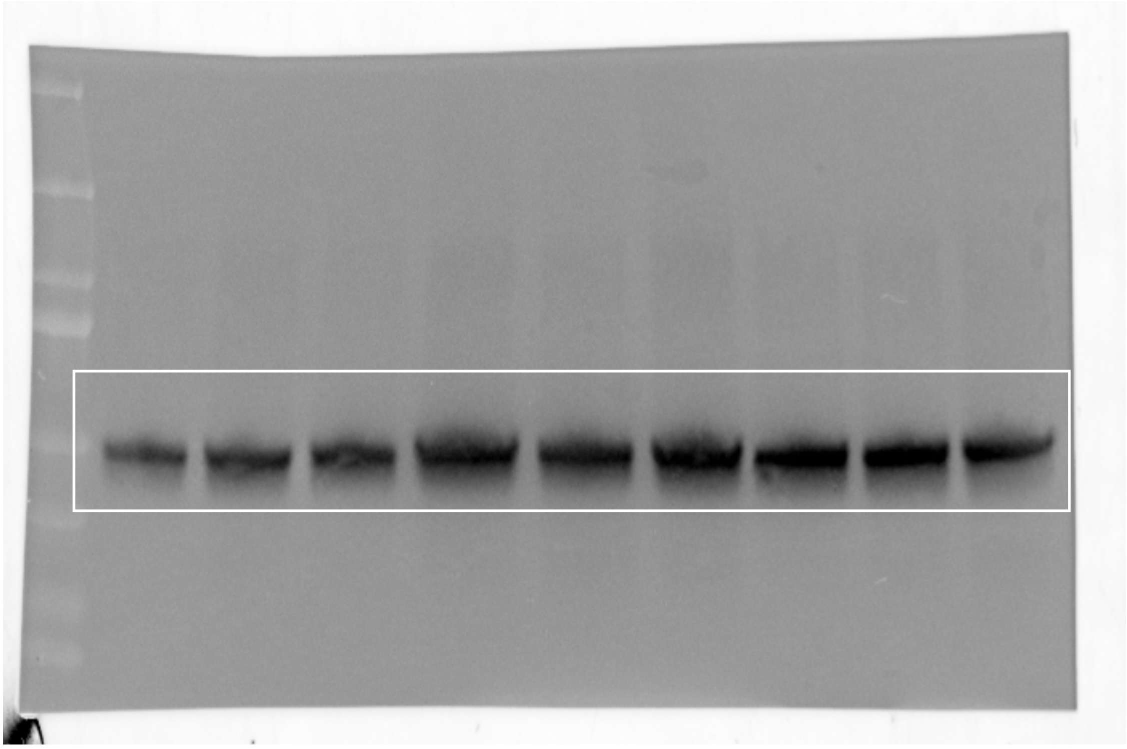

GSK3β

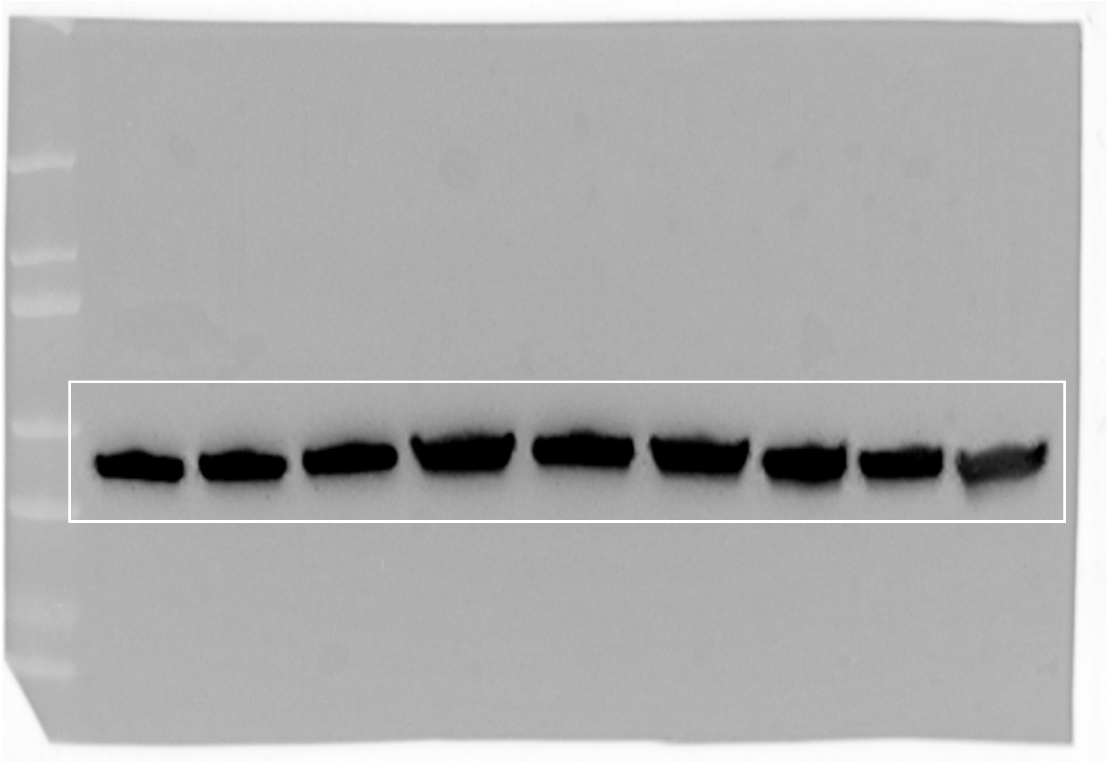

Actin
